# Successful Endovascular Management of Pseudoaneurysm following Transarterial Chemoembolization: A Case Report

**DOI:** 10.3390/medicina60050701

**Published:** 2024-04-25

**Authors:** Joo Yeon Jang, Tae Un Kim, Hwaseong Ryu, Ki Tae Yoon, Young Mi Hong, Ung Bae Jeon

**Affiliations:** 1 Department of Radiology, Pusan National University Yangsan Hospital, School of Medicine, Pusan National University, Yangsan 50612, Republic of Korea; jycj1008@hanmail.net (J.Y.J.); kimtaeun78@hanmail.net (T.U.K.); yocomeon@gmail.com (H.R.); 2 Department of Internal Medicine, Pusan National University Yangsan Hospital, School of Medicine, Pusan National University, Yangsan 50612, Republic of Korea; ktyoon@pusan.ac.kr (K.T.Y.); 00gurum@hanmail.net (Y.M.H.)

**Keywords:** aneurysm, chemoembolization, hemobilia, hepatic artery, N-Butyl-cyanoacrylate

## Abstract

*Background and Objectives*: Transarterial chemoembolization (TACE) is a widely accepted treatment for hepatocellular carcinoma (HCC). Regarding TACE, arterial injuries, such as hepatic artery spasm or dissection, can also occur, although pseudoaneurysms are rare. We report a case of pseudoaneurysm following TACE. *Materials and Methods*: A 78-year-old man had been undergoing TACE for HCC in segment 8 of the liver for the past 5 years, with the most recent TACE procedure performed approximately 1 month prior. He presented to the emergency department with melena that persisted for 5 days. Computed tomography revealed a pseudoaneurysm in the S8 hepatic artery with hemobilia. *Results*: the pseudoaneurysm was successfully treated by N-Butyl-cyanoacrylate glue embolization. *Conclusions*: In patients that have undergone TACE presenting with melena and hemobilia identified on CT, consideration of hepatic artery pseudoaneurysm is crucial. Such cases can be safely and effectively treated with endovascular managements.

## 1. Introduction

Transarterial chemoembolization (TACE) is a widely accepted treatment for hepatic tumors, particularly unresectable hepatocellular carcinoma (HCC). Although it is generally safe, TACE can lead to severe complications, including liver abscesses, liver failure, and non-target embolization [1]. Arterial injuries, such as hepatic artery spasm or dissection, can also occur, although pseudoaneurysms are rare [2]. Cases of hepatic artery pseudoaneurysm associated with TACE are exceedingly rare. Moreover, there are no cases, to the best of the authors’ knowledge, of pseudoaneurysm occurring as a late complication in patients who underwent only TACE. In this case report, we aim to present a case of hepatic artery pseudoaneurysm that occurred one month after TACE, and was successfully treated with N-Butyl-cyanoacrylate (NBCA) glue embolization.

## 2. Case Report

A 78-year-old man presented to the emergency department with melena that persisted for 5 days. He had a previous diagnosis of HCC in segment 8 of the liver, and had undergone five sessions of TACE over the past 5 years, with the most recent TACE procedure performed approximately 1 month prior. Upon admission, his vital signs were as follows: blood pressure, 80/50 mmHg; heart rate, 101 bpm; and temperature, 38.4 °C. Laboratory results on the same day indicated the following: hemoglobin levels, 10.7 g/dL; platelet count, 200 × 10^3^/μL; prothrombin time/international normalized ratio, 1.09; serum bilirubin levels, 5.6 mg/dL; albumin levels, 3 g/dL; aspartate aminotransferase levels, 106 IU/L; alanine aminotransferase levels, 69 IU/L; gamma-glutamyl transferase levels, 533 IU/L; amylase levels, 1011 IU/L; lipase levels, 3686 U/L; and high-sensitive C-reactive protein, 19.66 mg/dL. Clinically, biliary sepsis was suspected based on these findings.

**Figure 1:**
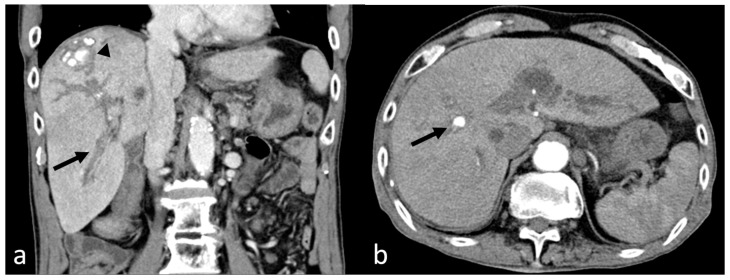
(**a**) The coronal computed tomography (CT) image reveals hemobilia in the right intrahepatic duct (arrow) and hepatocellular carcinoma (arrowhead) in liver segment 8, previously treated with transarterial chemoembolization (**b**) The axial CT image shows a pseudoaneurysm (arrow) in the hepatic artery of segment 8.

To differentiate whether the cause of melena was gastrointestinal bleeding, abdominal computed tomography (CT) was performed. However, active bleeding was not seen in the GI tract, and although the patient had liver cirrhosis, there were no signs of gastroesophageal varices. Instead, it revealed hemobilia in the anterior branches of the right intrahepatic duct, common bile duct, and gallbladder (Figure 1a). Additionally, a pseudoaneurysm was detected in the S8 hepatic artery (Figure 1b).

**Figure 2:**
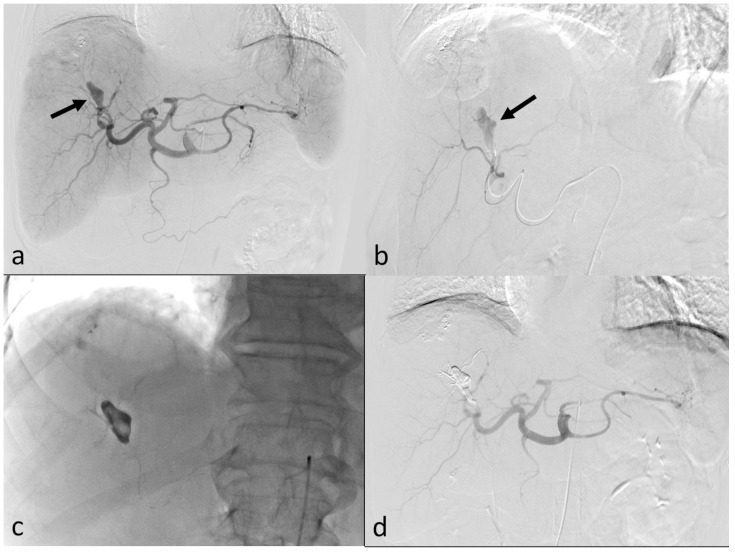
(**a**) Celiac angiography displays a fusiform-shaped pseudoaneurysm (arrow) originating from the segment 8 hepatic artery. (**b**) Hepatic arteriography with 45° left anterior oblique view shows the location of pseudoaneurysm (arrow) clearly. (**c**) The pseudoaneurysm was embolized with a mixture of lipiodol and N-Butyl-cyanoacrylate (NBCA) glue. (**d**) Following NBCA embolization, the pseudoaneurysm becomes undetectable on the final angiography.

Celiac arteriography using a 5F catheter (Rosch Hepatic; Cook, Bloomington, IN, USA) showed a fusiform-shaped pseudoaneurysm originating from segment 8 of the hepatic artery (Figure 2a). The shape of the pseudoaneurysm appeared to be different from that in the previous CT image, raising the suspicion of rupture. In fact, the pseudoaneurysm was not detected during the CT interpretation, so angiography was performed the day after the CT scan. Therefore, it is believed that the shape of the pseudoaneurysm changed during that time. Selective arteriography with 45-degree left anterior oblique view was performed through the hepatic artery using a microcatheter (Asahi Tellus; ASAHI INTECC Co., Ltd., Seto, Japan) and micro-guidewire (Asahi Meister; ASAHI INTECC Co., Ltd.). Selective arteriography confirmed that the pseudoaneurysm was located in the proximal S8 hepatic artery (Figure 2b). This artery was embolized with a mixture of 1.5 mL iodized oil (Lipiodol Ultrafluid; Andre Guerbet, Aulnay-sous-bois, France) and 0.5 mL n-butyl-2-cyanoacrylate (Histoacryl; B. Braun, Melsungen, Germany) glue (Figure 2c). Consequently, the pseudoaneurysm and extravasation disappeared on the final angiography (Figure 2d).

**Figure 3:**
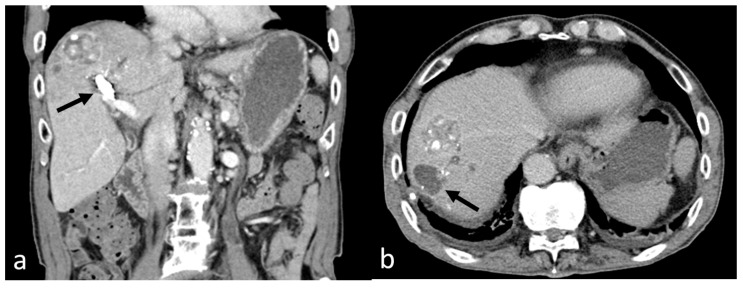
(**a**) The pseudoaneurysm was successfully embolized (arrow), and hemobilia was resolved as found on the follow-up computed tomography image. (**b**) However, several bilomas (arrow) were newly observed in segments 5 and 8 of the liver.

The following day, the serum bilirubin levels decreased to 2.8 mg/dL, and the amylase and lipase levels returned to normal. Approximately 6 days later, the pseudoaneurysm was successfully embolized, and the hemobilia was resolved as found on CT (Figure 3a). However, several new bilomas were observed in segments 5 and 8 of the liver (Figure 3b), which had resolved on a 4-month follow-up CT scan.

## 3. Discussion

TACE is a well-established and frequently used treatment for HCC. Despite its general safety, owing to the liver’s dual blood supply, TACE is associated with potential complications [3]. The most common complication is post-embolization syndrome, which is characterized by symptoms such as abdominal pain, nausea, and fever. However, in a subset of patients, more severe complications may arise, occurring in approximately 4–6% of cases. Moreover, complications include liver abscesses, liver failure, non-target embolization, intrahepatic biloma, cholecystitis, and biliary strictures [1].

The patient in this case presented with melena rather than the aforementioned symptoms. Upon performing a CT scan to identify the cause of melena, gastrointestinal bleeding was not observed, but hemobilia was detected. In fact, the clinical manifestation of hemobilia varies greatly depending on the rate of blood loss. Like our case, rapid arterial bleeding can result in a significant blood influx into the bile and, subsequently, into the duodenum, leading to symptoms such as hematemesis or melena. Severe cases of massive bleeding may also cause abrupt dilation of the extrahepatic bile ducts, resulting in colic pain or even shock and potential fatality [4].

However, diagnosis of hemobilia often occurs belatedly, and may be inadequately managed for prolonged periods. Therefore, physicians must be cognizant of the diverse etiologies and presentations of hemobilia, including the less common ones [4]. Suspecting hemobilia becomes paramount once more common sources of gastrointestinal bleeding are excluded using esophagogastroduodenoscopy. Nonetheless, when hemobilia is suspected, selective angiography of the hepatic artery remains the most accurate diagnostic tool for identifying the bleeding source. It often aids in defining and localizing arterial lesions, such as arterio-biliary fistulas, arterio-portal fistulas, and pseudoaneurysm [5,6].

To date, among the various therapeutic options, transcatheter arterial embolization (TAE) is regarded as the preferred treatment over surgery due to its safety, minimally invasive nature, and the ability to integrate diagnostic angiography with interventional procedures [5,6]. Super-selective catheterization of the bleeding source’s feeding vessel, followed by embolization using absorbable particles, appears to be the most efficacious approach. Furthermore, super-selective embolization is important for minimal liver damage. In situations where catheterization of a peripheral branch is not feasible, or when immediate intervention is warranted due to the patient’s clinical status, central embolization of the main hepatic artery or one of its major branches is indicated [4]. In cases involving pseudoaneurysms, coils should be precisely positioned just distal to the arterial injury, and extended across the lesion to bridge the neck of the pseudoaneurysm [5].

In our patient, a pseudoaneurysm was also located in the proximal S8 hepatic artery, necessitating central embolization. Although superselection was attempted, given the central location of the pseudoaneurysm and the use of histoacryl rather than temporary embolic material, it is necessary to assess for liver damage. Moreover, as the bile duct receives blood supply from the hepatic artery, it is essential to confirm the presence of biliary complications after embolizing the hepatic artery with permanent embolic material such as histoacryl. Therefore, prior to discharge, a CT scan was performed. Indeed, in the patient’s case, new bilomas had formed, which was found to have resolved on follow-up CT scans approximately four months later. For reference, coils can also be used as embolic material for pseudoaneurysm. However, for pseudoaneurysms, it is believed that filling them completely with histoacryl is more advantageous for embolization, as the arterial walls are not fully intact. Additionally, using coils can prolong procedure time, and may affect the interpretation of follow-up imaging due to metallic artifacts. Therefore, in our institution, we primarily use histoacryl for embolization in cases of pseudoaneurysms.

TACE-related vascular complications include hepatic artery injuries, such as occlusion and dissection, collateralization within the intrahepatic and extrahepatic regions, aneurysm formation, and pseudoaneurysm development. Dissection is typically attributed to guidewire-induced injuries, whereas hepatic artery stenosis, occlusion, and aneurysm formation are associated with irritation caused by chemotherapeutic drugs and gelatin sponge particles. Catheter and guidewire-related injuries contribute to the development of aneurysmal changes, including pseudoaneurysm formation [7]. Lucatelli et al. [8] conducted a study comparing balloon-occluded TACE and DEB TACE. In that study, the authors reported that adverse events were observed without a significant difference between B-TACE and DEM-TACE. Specifically, a pseudoaneurysm was noted following a B-TACE procedure. However, pseudoaneurysms observed in the B-TACE subgroup occurred during the initial learning curve of balloon microcatheter usage, within the first five cases. It is suggested that B-TACE itself may not necessarily increase the risk of pseudoaneurysm. To the best of the authors’ knowledge, there have been no studies investigating the association between TACE techniques and the frequency of pseudoaneurysm occurrence.

Instead, pseudoaneurysms occurring after TACE have only been reported in a few cases. One such case was reported by Sueyoshi et al. [9], who described a pseudoaneurysm arising from guidewire manipulation during TACE. Recently, Atay and Ozdemir reported an unusual case of a post-TACE pseudoaneurysm [2]. The pseudoaneurysm was identified on the final angiography and effectively managed through endovascular treatment using pushable microcoils.

Intrahepatic pseudoaneurysms can also occur as delayed complications, as was observed in our case. Hasegawa et al. [10] reported a case in which a pseudoaneurysm with hemobilia manifested 38 days following concurrent TACE and radiofrequency ablation. However, no case of pseudoaneurysm developing as a late complication of TACE has been reported to date. Although the mechanism of intrahepatic aneurysm formation after TACE remains controversial, it may be attributed to arterial wall fragility caused by inflammatory and reparative changes after TACE, increased pressure from the blood flow during recanalization, or turbulent flow caused by an organized thrombus [11].

In real-world clinical scenarios, when a patient with liver cirrhosis undergoes a CT scan due to melena, a thorough examination is carried out to determine the presence of arterial active bleeding in the stomach or duodenum, or the existence of gastroesophageal varices. Surprisingly, the hepatic artery pseudoaneurysm was overlooked during the CT interpretation in this patient. In cases where a patient with liver cirrhosis presents with melena, particularly following recent TACE, it is uncommon but crucial to consider the possibility of hepatic artery injury. This suspicion is heightened, especially when hemobilia is concurrently present.

## 4. Conclusions

In conclusion, although hepatic artery pseudoaneurysms are rare, they can occur as a late complication of TACE. Therefore, in patients that have undergone TACE presenting with melena and hemobilia identified on CT, consideration of iatrogenic arterial injury is crucial. Such cases can be safely and effectively treated with endovascular procedures.

## Data Availability

The data presented in this study are available upon request from the corresponding author.

## References

[1] Cammà C., Schepis F., Orlando A., Albanese M., Shahied L., Trevisani F., Andreone P., Craxi A., Cottone M. (2002). Transarterial chemoembolization for unresectable hepatocellular carcinoma: Meta-analysis of randomized controlled trials. Radiology.

[2] Atay M., Ozdemir H. (2022). An Unusual Complication of Transarterial Chemoembolization of Hepatocellular Carcinoma; Pseudoaneurysm: A Case Report. Curr. Med. Imaging.

[3] Shin S.W. (2009). The current practice of transarterial chemoembolization for the treatment of hepatocellular carcinoma. Korean J. Radiol..

[4] Libra F., Santonocito S., Falsaperla D., Failla G., Palmucci S., Basile A. (2021). Endovascular treatment of a rare case of haemobilia caused by both pseudoaneurysm and a giant hepatic haemangioma. Radiol. Case Rep..

[5] Srivastava D.N., Sharma S., Pal S., Thulkar S., Seith A., Bandhu S., Pande G.K., Sahni P. (2006). Transcatheter arterial embolization in the management of hemobilia. Abdom. Imaging.

[6] Birth M., Ortlepp J., Bontikous S., Amthor M., Weiser H.-F., Bruch H.-P. (2000). Intermittent activity-induced hemobilia caused by liver hemangioma. Dig. Surg..

[7] Maeda N., Osuga K., Mikami K., Higashihara H., Onishi H., Nakaya Y., Tatsumi M., Hori M., Kim T., Tomoda K. (2008). Angiographic evaluation of hepatic arterial damage after transarterial chemoembolization for hepatocellular carcinoma. Radiat. Med..

[8] Lucatelli P., De Rubeis G., Rocco B., Basilico F., Cannavale A., Abbatecola A., Nardis P.G., Corona M., Brozzetti S., Catalano C. (2021). Balloon occluded TACE (B-TACE) vs DEM-TACE for HCC: A single center retrospective case control study. BMC Gastroenterol..

[9] Sueyoshi E., Hayashida T., Sakamoto I., Uetani M. (2010). Vascular complications of hepatic artery after transcatheter arterial chemoembolization in patients with hepatocellular carcinoma. Am. J. Roentgenol..

[10] Hasegawa T., Inaba Y., Takahashi M., Chatani S., Dejima I., Tsukamoto H., Murata S., Kato M., Sato Y., Yamaura H. (2019). Pseudoaneurysm formation and hemobilia as late complications after transarterial chemoembolization and rad iofrequency ablation for hepatocellular carcinoma. Interv. Radiol..

[11] Sakamoto I., Aso N., Nagaoki K., Matsuoka Y., Uetani M., Ashizawa K., Iwanaga S., Mori M., Morikawa M., Fukuda T. (1998). Complications associated with transcatheter arterial embolization for hepatic tumors. Radiographics.

